# Mortality prediction in women with corpus uteri cancer in Brazil: a 21-year analysis

**DOI:** 10.3332/ecancer.2020.1029

**Published:** 2020-05-04

**Authors:** Diego Bessa Dantas, Lucio Flávio Garcia Rodrigues, Fabiana de Campos Gomes, João Simão de Melo-Neto

**Affiliations:** 1 Federal University of Pará (UFPA), Belém, PA, Brazil; 2 School of Medicine of São José do Rio Preto (FAMERP), São José do Rio Preto, SP, Brazil

**Keywords:** Brazil, uterine neoplasms, mortality, epidemiology

## Abstract

Mortality data obtained from the Mortality Information System identified a total of 19,499 deaths in women caused by corpus uteri cancer in Brazil. However, the association between mortality and sociodemographic factors in these women is not fully understood. A study based on the secondary data on deaths caused by corpus uteri cancer recorded in the SIM-DATASUS was conducted. Deaths reported from 1996 to 2016 in the health information system were included. Sociodemographic factors were analysed to determine their association with mortality. Low schooling is highly associated with mortality in all administrative regions. Advanced age, race and marital status have specific association with mortality for the different geographic regions. Black, Brown and Indigenous women with low schooling and of advanced age are highly associated with mortality. Brown, White and Black women of advanced age had the highest corpus uteri cancer related mortality rates. Women with low schooling who died of corpus uteri cancer were either single or widows. The marital status of Black, White and Brown women aged <59 years was single. The sociodemographic factors that predict mortality in women with corpus uteri cancer in Brazil were presented and can be used to guide public health.

## Introduction

Tumours of the corpus uteri are divided into the following two main groups: endometrial tumours and mesenchymal tumours. The former are common gynaecological diseases, whereas the latter manifest more aggressively and are rarer with worse prognosis than the former. Endometrioid adenocarcinomas and carcinosarcomas or leiomyosarcomas, considering clear types of tumours alone, are the most common types of endometrial tumours and mesenchymal tumours, respectively [1].

The proportion of adenocarcinomas accounts for greater than 80% of all corpus uteri cancers in all countries studied, except in Brazil (74.1%). In Brazil, the proportion of unspecified morphology is comparatively high (13.1%), and the proportion of sarcoma is low and is approximately 1.2%–5.1% of all corpus uteri cancers [2].

Approximately, 95% of uterine malignancies are endometrial carcinoma [3]. Worldwide, the incidence of endometrial cancer is rapidly increasing, with the highest disease burden reported in North America and Western Europe [4]. However, the epidemiological data associated with mortality in Brazil are unknown.

The onset of corpus utreri cancer is usually in postmenopausal women. Its occurrence and mortality are highly associated with overweight and obese women [5]. Additionally, understanding the association between the sociodemographic factors (geographic region, age, marital status, race and schooling) and mortality caused by corpus uteri cancers aids in the development of public policies aimed at the most vulnerable population.

Mortality data obtained from the Mortality Information System (SIM) of the Brazilian Ministry of Health, available on the DATASUS website with annual data collected from 1996 to 2016, identified a total of 19,499 deaths of women caused by corpus uteri cancer in Brazil [6]. Comparative studies have shown an association between mortality prediction, survival and sociodemographic factors in women with corpus uteri cancer, supporting the need to increase the number of studies that present consistent data on the subjects [7].

This study aimed to analyse the sociodemographic factors that predict mortality in corpus uteri cancer in Brazil. Specifically, the sociodemographic factors (geographic regions, age, race/ethnicity and schooling) will be evaluated to determine their association with mortality from 1996 to 2016.

## Methods

### Ethics

This study analyses secondary data available in the DATASUS. The data are publicised with unrestricted use and access. Ethical assessment of the research ethics committee is not required according to the terms of the National Health Council Resolution No. 466 of December 12, 2012.

### Type of study

An analytic, descriptive and retrospective study based on secondary data on deaths caused by corpus uteri cancer recorded in the SIM of the Ministry of Health of Brazil was conducted.

### Database

The SIM is a secondary database available in the Informatics Department of the Brazilian National Health System (DATASUS) of the Ministry of Health [8]. Deaths reported from 1996 to 2016 in Brazil in the health information system, and classified by the International Classification of Diseases [9], defined according to the 10th revision by code C54 (43), were included.

### Study variables

Geographic regions, age, marital status, race/ethnicity and educational attainment were considered the sociodemographic factors. These factors were further categorised as follows: geographic regions (North, Northeast, Midwest, South and Southeast), race/ethnicity (Brown, White, Black, Yellow and Indigenous), age (less than 19, 20–29, 30–39, 40–49, 50–59, 60–69, 70–79 and greater than 80 years), marital status (single, married, widowed and divorced) and schooling (no schooling greater than 12 years).

### Statistical analysis

The data were submitted for descriptive and inferential analysis. For the description of data, absolute and relative frequencies were used. Age-period-cohort (APC) analysis using a suitable model that accounts for the identification problem to discern variations in mortality due to independent effects of age groups, calendar time periods of death and birth cohorts was performed. For all analysed variables in this study, the following functions have been estimated: net drift (overall annual percentage change in accordance with calendar period and birth cohort); local drifts (annual percentage changes for each age group in accordance with calendar period and birth cohort); all age deviations (fitted longitudinal and cross-sectional age curves are log-linear); all period deviations (fitted temporal trends and period rate ratios are log-linear); all cohorts deviations (cohort rate ratios are log-linear and all local drifts equal the net drift); and all period (or cohort) rate ratios (RR) (age incidence pattern in every period (or cohort)). Wald test was used to verify difference significative, being considered *p* < 0.05. We obtained these estimable parameters by the APC Web Tool (Biostatistics Branch, National Cancer Institute, Bethesda, MD, USA) [10]. The chi-squared test with Yates’s correction as used to analyse the association between sociodemographic factors and mortality caused by corpus uteri cancer. To quantify the level of association, odds ratios with 95% confidence intervals (95% CI) for the occurrences of death in women with corpus uteri cancer were used.

## Results

**Figure 1.**
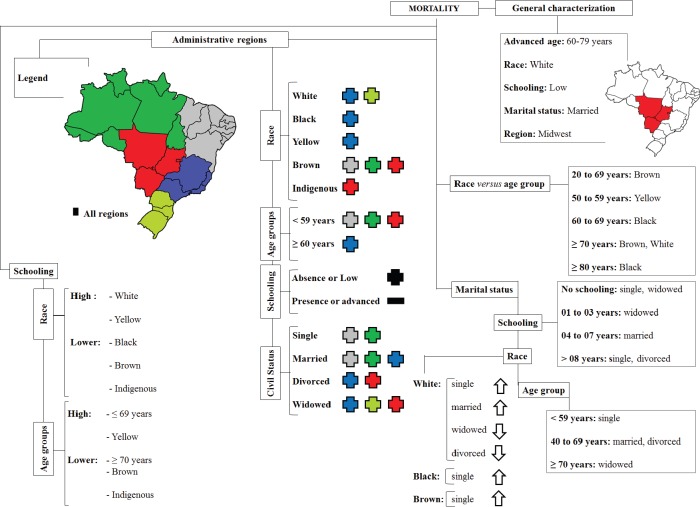
Diagram showing the sociodemographic factors associated with mortality caused by corpus uteri cancer.

The highest number of deaths from uterine cancer was observed in women with the following characteristics: aged 60 to 79 years (59.02%), belonging to the White race (61.44%), with low education ≤3 (31.58%), married (34.84%) or widowed (33.54%) and reside in the Midwest (56.33%) of Brazil. The results are shown in Figure 1.

### APC analysis

**Figure 2.**
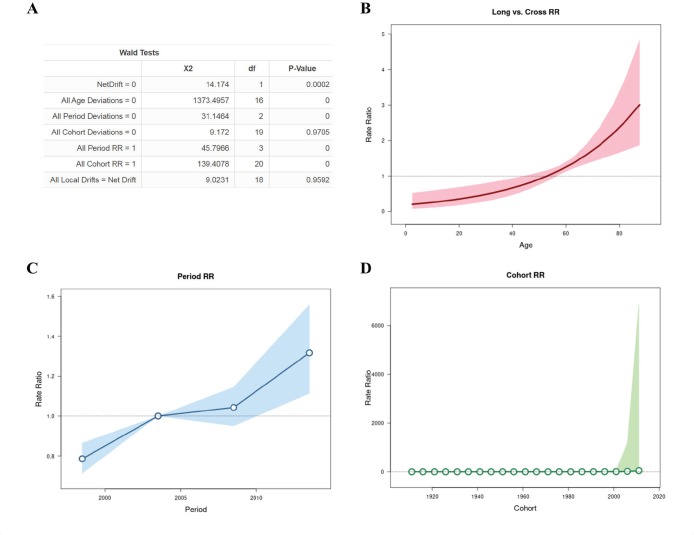
APC analysis with Wald test (A), all age deviations (B), period rate ratios (RR) (C) and cohort (RR) (D) .

The results obtained in the analysis of the APC are seen in Figure 2. During the period of 1996 to 2016, the net drift, that represented annual percentage change of the expected age-adjusted rates, was 3.237% (95% CI: 1.539–4.964) per year. Local drift values and cohorts’ deviations are not statistically significant. All age deviations demonstrated that there is greater risk of progressing to death with advancing age in relation to the younger individuals progressively until the last years of life, with a greater peak after 80 years of age (Figure 2B). On the other hand, younger women had a lower risk with RR <1 up to 38 years of age. All period deviations demonstrated that fitted temporal trends and period RR (Figure 2C) are log-linear, indicating that age pattern of patients that death in every period with increase in recent years. All cohort RR indicated an age incidence pattern in every birth cohort (Figure 2D).

### Administrative regions versus race, age group, schooling and marital status

The association between administrative regions and race, age group, schooling and marital status is presented in Table 1.

According to the sociodemographic factors, the association between geographic region and race and mortality was as follows: White women from the South and Southeast regions, Black and Yellow women from the Southeast region, and Brown women from the North, Northeast, and Midwest regions had six times higher chance of mortality than those from the rest of the regions. Indigenous women from the Midwest region were highly associated with mortality, with five times higher chance of mortality compared to those from the rest of the regions.

Hence, an association between women’s’ geographic region and age and mortality caused by corpus uteri cancer was observed, and from these data it was, women aged <19 years in the Northeast region; women aged 50–59 years in the North, Northern and Midwest regions; and women aged 60–69, 70–79 and greater than 80 years in the Southeast region were highly associated with mortality.

Regarding the level of schooling, women from the North, Northeast, and Midwest regions with no schooling, women from the South and Southeast regions with 1–7 years of experience in schooling, and women from the Southeast region with 8–11 years and ≥12 years of experience in schooling were highly associated with mortality caused by corpus uteri cancer. However, women who presently study (North, Northeast and Midwest regions) or had advanced schooling (South region) exhibited lower odds of mortality. Interestingly, there were lower odds of mortality in the Southeast region.

According to the data on the marital status of women, single and married women from the North and the Northeast regions, married and widowed women from the South region, widowed and divorced women from the Southeast region and married and divorced women from the Midwest region were highly associated with mortality caused by corpus uteri cancer.

### Race versus age group

The association between race and age group and mortality caused by corpus uteri cancer is presented in Table 2. White women older than 70 years, Black women aged 60–69 years, Yellow women aged 50–59 years and Brown women aged 20–69 years were highly associated with mortality.

### Schooling versus race and age group

White and Yellow women with high education and Black, Brown and Indigenous women with low education were highly associated with mortality. White women with low education and Black with high educational level had lower odds of mortality. Indigenous women with high education and Yellow women with low education had no association with mortality. Women aged ≤69 years with high level of education and women aged ≥70 years with low level of education were highly associated with mortality.

### Marital status versus race, age group and schooling

The association between marital status, race, age group and schooling and mortality caused by corpus uteri cancer is presented in Table 2. Single and widowed women with no schooling and widowed women who had 1–3 years of experience in schooling, married women with 4–7 years of experience in schooling, and women with ‘8–11 years’ and ‘greater than 12 years’ of experience in schooling were highly associated with mortality.

The association between age group and mortality caused by corpus uteri cancer was as follows: single women aged <59 years were highly associated with mortality, and women aged <19 years had 59 times higher chance of mortality. Married and divorced women aged 40–69 years were highly associated with mortality. Moreover, widowed women aged ≥70 years were associated with mortality, with women aged greater than 80 years having five times higher chance of mortality.

Additionally, White and single and married women exhibited lower odds of mortality, while widowed and divorced women were positively associated with mortality. Moreover, Black and Brown single women were highly associated with mortality.

## Discussion

Corpus uteri cancer is a very common gynaecological malignancy, especially in high-income countries. Although the overall prognosis is relatively good, high-grade corpus uteri cancer tends to recur. Recurrence needs to be prevented since the prognosis for cancer recurrence is worse than the initial cancer. This study analysed the sociodemographic factors that predict mortality caused by corpus uteri cancer in Brazil [11]. Specifically, the sociodemographic factors (geographic regions, age, race and schooling) were evaluated to determine their association with mortality from 1996 to 2016.

The results of APC analysis revealed that mortality is higher with increasing age. Black women aged 60–69 years, Yellow women aged 50–59 years, and Brown women aged 20–69 years were highly associated with mortality, with results showing that higher chance of mortality was noted even in younger women. The association between elderly women and mortality caused by corpus uteri cancer is well understood in the literature, showing a higher risk mortality in elderly women in relation to younger women [12].

According to a study using the data from Brazil, Black women presented a higher percentage of corpus uteri cancer progression or recurrence compared to non-Black women, and all of these women benefited from the public health services offered, a common characteristic that makes this group homogeneous [13].

Cancer health differences are often described as the unequal burden of cancer deaths in one racial/ethnic group compared to another. For example, the National Cancer Statistics in the USA shows that death from 9 out of the top 10 cancers in men and women is mostly observed in Blacks. Considering that there is no association between genetic and biological variances for these differences, it is possible to associate these results with the unequal distribution of the social determinants of health as the primary cause of cancer differences [14].

It was confirmed that Black, Brown and Indigenous women with low schooling have a greater association with mortality and White and Yellow women with a high level of schooling. It can be hypothesised that the low schooling group has greater difficulty in accessing healthcare services compared to the other groups. Low educational levels can lead to low health literacy; hence, women with high educational level are able to access, understand, and act on complex health information and communicate with healthcare personnel [14]. However, in relation to the group with a high level of schooling, according to epidemiological studies, it is possible that their greater purchasing power is highly associated with obesity [15]. Hence, the association between obesity and cancer has to be considered [16].

According to the presented results on marital status, there is a lower association between mortality and married women compared to other marital statuses, supporting other studies [17, 18] that associate single women, including widows, with significantly higher risk of metastatic cancer, resulting even in death, than married women. The importance of this study is that it highlights the consistent and substantial impact that marriage status has on cancer. The general hypothesis between these studies was that married women have a greater social support system than single women, which improves their overall health maintenance, including medication adherence [19].

It was observed that in the North and Northeast regions, mortality was higher in Brown women aged less than 60 years, with emphasis on the association in Indigenous women living in the Midwest region. These women have difficulty accessing the health policies in the country, mainly due to geographical and cultural barriers [20].

These results are possibly associated with women in these regions having higher difficulty accessing the oncological treatment centres compared to the South and Southeast regions, where the highest number of mortality is observed in women aged greater than 60 years [21]. The structural differences between the different regions in Brazil in the public health system lead women to migrate to search for better conditions in treating their diseases. In addition to the discomfort experienced by women, care is focused on large healthcare centres, causing an overload on the current healthcare capacity [22].

The results of the analysis support the initial hypothesis that the interval between cancer diagnosis and early treatment is longer for women with vulnerable social characteristics, regardless of the stage of the disease, compared to women with no vulnerable social characteristics. There is a clear consensus in the literature that the shorter the interval between diagnosis and treatment, the better the prognosis and patient survival. Immediate action is essential to the effectiveness of treatment in more advanced stages of the disease or patient comfort in palliative treatment [23].

Understanding the association between sociodemographic factors and mortality caused by corpus uteri cancer is essential for the development of public policies worldwide, but in Brazil, similar to other developing countries, it is necessary to recognise that there are limitations on the quality of data collection. A very high number of unknown or unreported data that greatly undermine the reliability of the analysis performed in studies using secondary banks are noted. On the contrary, the number of unknown data has declined over the years. Another limitation is characterised by the change of terms and items in the collection worksheets, reducing the standardisation in the collection and data releases in the platform [24].

## Conclusion

In this study, we found that the sociodemographic factors of race, age, schooling, marital status and geographic regions present specific characteristics that predict mortality in women with corpus uteri cancer in Brazil. These findings can be used to review or develop new public health guidelines and policies. Thus, there is a need to improve the existing public policies to prevent death caused by corpus uteri cancer, especially for the most vulnerable population with less social support and greater difficulty in accessing oncological healthcare services.

## Conflict of interest

The authors have no conflicts of interest to disclose.

## Authors’ contributions

The authors participated in all the stages of the study.

## Funding statement

No funding was received for this work.

## Figures and Tables

**Table 1.** Association between geographic regions and race, age group, schooling and marital status.﻿

Geographic regions					
	North	Northeast	Southeast	South	Midwest
**Race**					
White	*N* = 163 *p* = 0.0001 OR = 0.188 95% CI: 0.156–0.227	*N* = 1,133 *p* = 0.0001 OR = 0.217 95% CI: 0.200–0.236	*N* = 7,279 *p* = 0.0001 OR = 1.686 95% CI: 1.582–1.798	*N* = 2,919 *p* = 0.0001 OR = 4.955 95% CI: 4.407–5.572	*N* = 488 *p* = 0.0001 OR = 0.583 95% CI: 0.508–0.670
Black	*N* = 33 *p* = 0.099 OR = 0.731 95% CI: 0.512–1.044	*N* = 212 *p* = 0.067 OR = 0.865 95% CI: 0.744–1.007	*N* = 941 *p* = 0.0001 OR = 1.577 95% CI: 1.406–1.768	*N* = 167 *p* = 0.0001 OR = 0.565 95% CI: 0.479–0.667	*N* = 61 *p* = 0.289 OR = 0.858 95% CI: 0.658–1.120
Yellow	*N* = 00 *p* = 0.018 OR = 0.075 95% CI: 0.004–1.220	*N* = 23 *p* = 0.046 OR = 0.631 95% CI: 0.408–0.976	*N* = 144 *p* = 0.0001 OR = 1.880 95% CI: 1.387–2.549	*N* = 23 *p* = 0.010 OR = 0.557 95% CI: 0.360–0.862	*N* = 13 *p* = 0.418 OR = 1.323 95% CI: 0.751–2.330
Brown	*N* = 346 *p* = 0.0001 OR = 6.498 95% CI: 5.436–7.768	*N* = 1,575 *p* = 0.0001 OR = 5.913 95% CI: 5.432–6.435	*N* = 1,584 *p* = 0.0001 OR = 0.419 95% CI: 0.389–0.450	*N* = 148 *p* = 0.0001 OR = 0.131 95% CI: 0.110–0.155	*N* = 301 *p* = 0.0001 OR = 1.899 95% CI: 1.644–2.195
Indigenous	*N* = 02 *p* = 0.172 OR = 4.184 95% CI: 0.954–18.345	*N* = 03 *p* = 0.819 OR = 1.063 95% CI: 0.305–3.704	*N* = 5 *p* = 0.043 OR = 0.318 95% CI: 0.112–0.904	*N* = 03 *p* = 0.829 OR = 0.940 95% CI: 0.270–3.274	*N* = 04 *p* = 0.002 OR = 5.950 95% CI: 1.936–18.288
Others*	*N* = 43	*N* = 410	*N* = 1,032	*N* = 311	*N* = 133
**Age**					
≤19 age	*N* = 00 *p* = 0.618 OR = 1.892 95% CI: 0.109–32.840	*N* = 04 *p* = 0.046 OR = 4.819 95% CI: 1.204–19.278	*N* = 03 *p* = 0.472 OR = 0.464 95% CI: 0.111–1.945	*N* = 01 *p* = 0.975 OR = 0.636 95% CI: 0.078–5.177	*N* = 00 *p* = 0.510 OR = 1.088 95% CI: 0.062–18.873
20 to 29 age	*N* = 10 *p* = 0.0001 OR = 5.022 95% CI: 2.567–9.824	*N* = 18 *p* = 0.158 OR = 1.523 95% CI: 0.895–2.590	*N* = 31 *p* = 0.012 OR = 0.544 95% CI: 0.343–0.863	*N* = 14 *p* = 0.985 OR = 1.040 95% CI: 0.580–1,863	*N* = 02 *p* = 0.480 OR = 0.506 95% CI: 0.124–2.065
30 to 39 age	*N* = 20 *p* = 0.001 OR = 2.209 95% CI: 1.394–3.501	*N* = 102 *p* = 0.0001 OR = 2.324 95% CI: 1.830–2.950	*N* = 104 *p* = 0.0001 OR = 0.372 95% CI: 0.294–0.471	*N* = 68 *p* = 0.168 OR = 1.221 95% CI: 0.931–1.601	*N* = 23 *p* = 0.108 OR = 1.458 95% CI: 0.949–2.240
40 to 49 age	*N* = 82 *p* = 0.0001 OR = 2.783 95% CI: 2.186–3.543	*N* = 260 *p* = 0.0001 OR = 1.486 95% CI: 1.287–1.716	*N* = 470 *p* = 0.0001 OR = 0.537 95% CI: 0.475–0.606	*N* = 220 *p* = 0.281 OR = 1.090 95% CI: 0.936–1.269	*N* = 92 *p* = 0.0001 OR = 1.716 95% CI: 1.372–2.145
50 to 59 age	N = 134 p = 0.0001 OR = 1.519 95% CI: 1.248–1.849	N = 623 p = 0.0004 OR = 1.193 95% CI: 1.083–1.314	N = 1,662 p = 0.0001 OR = 0.798 95% CI: 0.740–0.861	N = 606 p = 0.4121 OR = 1.042 95% CI: 0.946–1.148	N = 190 p = 0.030 OR = 1.200 95% CI: 1.020–1.413
60 to 69 age	*N* = 185 *p* = 0.527 OR = 1.063 95% CI: 0.890–1.268	*N* = 919 *p* = 0.0001 OR = 0.847 95% CI: 0.779–0.920	*N* = 3,424 *p* = 0.001 OR = 1.106 95% CI: 1.040–1.177	*N* = 1,049 *p* = 0.214 OR = 0.950 95% CI: 0.877–1.028	*N* = 319 *p* = 0.249 OR = 1.086 95% CI: 0.947–1.245
70 to 79 age	*N* = 99 *p* = 0.0001 OR = 0.492 95% CI: 0.395–0.612	*N* = 869 *p* = 0.0001 OR = 0.840 95% CI: 0.772–0.914	*N* = 3,388 *p* = 0.0001 OR = 1.258 95% CI: 1.181–1.340	*N* = 1,016 *p* = 0.615 OR = 0.978 95% CI: 0.903–1.060	*N* = 243 *p* = 0.001 OR = 0.785 95% CI: 0.677–0.910
≥80 age	*N* = 57 *p* = 0.0001 OR = 0.531 95% CI: 0.403–0.699	*N* = 557 *p* = 0.989 OR = 0.999 95% CI: 0.9043–1.104	*N* = 1,899 *p* = 0.004 OR = 1.117 95% CI: 1.035–1.206	*N* = 597 *p* = 0.886 OR = 1.008 95% CI: 0.914–1.111	*N* = 130 *p* = 0.002 OR = 0.739 95% CI: 0.612–0.893
Others*	*N* = 00	*N* = 04	*N* = 04	*N* = 00	*N* = 01
**Schooling**					
No schooling	*N* = 111 *p* = 0.0002 OR = 1.523 95% CI: 1.227–1.890	*N* = 695 *p* = 0.0001 OR = 2.860 95% CI: 2.577–3.174	*N* = 835 *p* = 0.0001 OR = 0.431 95% CI: 0.392–0.473	*N* = 379 *p* = 0.235 OR = 0.928 95% CI: 0.822–1.046	*N* = 156 *p* = 0.0001 OR = 1.498 95% CI: 1.247–1.800
01 to 03 years	*N* = 123 *p* = 0.021 OR = 0.781 95% CI: 0.635–0.960	*N* = 618 *p* = 0.005 OR = 0.815 95% CI: 0.782–0.956	*N* = 2,285 *p* = 0.019 OR = 1.093 95% CI: 1.015–1.178	*N* = 774 *p* = 0.037 OR = 1.106 95% CI: 1.006–1.215	*N* = 184 *p* = 0.034 OR = 0.828 95% CI: 0.697–0.983
04 to 07 years	*N* = 122 *p* = 0.516 OR = 0.928 95% CI: 0.754–1.142	*N* = 431 *p* = 0.0001 OR = 0.618 95% CI: 0.552–0.691	*N* = 2,084 *p* = 0.0001 OR = 1.227 95% CI: 1.135–1.326	*N* = 697 *p* = 0.006 OR = 1.147 95% CI: 1.041–1.264	*N* = 167 *p* = 0.143 OR = 0.872 95% CI: 0.730–1.042
08 to 11 years	*N* = 83 *p* = 0.940 OR = 1.016 95% CI: 0.800–1.292	*N* = 326 *p* = 0.001 OR = 0.815 95% CI: 0.718–0.926	*N* = 1,360 *p* = 0.0001 OR = 1.295 95% CI: 1.180–1.421	*N* = 347 *p* = 0.0003 OR = 0.793 95% CI: 0.700–0.897	*N* = 111 *p* = 0.557 OR = 0.934 95% CI: 0.759–1.150
≥ 12 years	*N* = 60 *p* = 0.929 OR = 1.022 95% CI: 0.776–1.346	*N* = 227 *p* = 0.002 OR = 0.790 95% CI: 0.681–0.916	*N* = 964 *p* = 0.0002 OR = 1.227 95% CI: 1.104–1.365	*N* = 258 *p* = 0.015 OR = 0.838 95% CI: 0.728–0.965	*N* = 92 *p* = 0.385 OR = 1.112 95% CI: 0.888–1.393
Others*	*N* = 88	*N* = 1,059	*N* = 3,457	*N* = 1,116	*N* = 290
**Marital status**					
Single	*N* = 159 *p* = 0.0001 OR = 1.490 95% CI: 1.234–1.799	*N* = 961 *p* = 0.0001 OR = 1.768 95% CI: 1.623–1.926	*N* = 2,211 *p* = 0.0001 OR = 0.829 95% CI: 0.773–0.888	*N* = 617 *p* = 0.0001 OR = 0.730 95% CI: 0.664–0.802	*N* = 181 *p* = 0.042 OR = 0.840 95% CI: 0.711–0.992
Married	N = 235 p = 0.0006 OR = 1.361 95% CI: 1.145–1.619	N = 1,085 p = 0.0001 OR = 1.177 95% CI: 1.086–1.275	N = 3,777 p = 0.001 OR = 0.905 95% CI: 0.852–0.961	N = 1,305 p = 0.032 OR = 1.088 95% CI: 1.008–1.174	N = 392 p = 0.0003 OR = 1.284 95% CI: 1.123–1.469
Widowed	*N* = 126 *p* = 0.0001 OR = 0.554 95% CI: 0.453–0.678	*N* = 901 *p* = 0.0001 OR = 0.723 95% CI: 0.664–0.786	*N* = 3,958 *p* = 0.0001 OR = 1.231 95% CI: 1.165–1.317	*N* = 1,284 *p* = 0.001 OR = 1.133 95% CI: 1.049–1.224	*N* = 272 *p* = 0.0001 OR = 0.752 95% CI: 0.650–0.869
Divorced	*N* = 16 *p* = 0.007 OR = 0.496 95% CI: 0.300–0.819	*N* = 123 *p* = 0.0001 OR = 0.642 95% CI: 0.530–0.778	*N* = 642 *p* = 0.038 OR = 1.145 95% CI: 1.009–1.299	*N* = 204 *p* = 0.550 OR = 1.052 95% CI: 0.899–1.232	*N* = 81 *p* = 0.0001 OR = 1.617 95% CI: 1.276–2.050
Others*	*N* = 51	*N* = 286	*N* = 397	*N* = 161	*N* = 74

* Category not defined.

**Table 2.** Association between marital status and race, age group and schooling.

**Race**						
	White	Black	Yellow	Brown	Indigenous	Others*
**Age**						
≤19 age	*N* = 04 *p* = 0.824 OR = 0.621 95% CI: 0.139–2.780	*N* = 01 *p* = 0.544 OR = 1.905 95% CI: 0.229–15.839	*N* = 00 *p* = 0.774 OR = 5.686 95% CI: 0.323–99.965	*N* = 02 *p* = 0.700 OR = 1.378 95% CI: 0.267–7.107	*N* = 00 *p* = 0.934 OR = 66.832 95% CI: 3.672–1216.5	*N* = 01
20 to 29 age	*N* = 36 *p* = 0.003 OR = 0.492 95% CI: 0.307–0.788	*N* = 05 *p* = 0.952 OR = 0.878 95% CI: 0.352–2.184	*N* = 00 *p* = 0.735 OR = 0.609 95% CI: 0.037–9.886	*N* = 28 *p* = 0.0008 OR = 2.305 95% CI: 1.427–3.724	*N* = 01 *p* = 0.095 OR = 15.842 95% CI: 2.071–121.18	*N* = 05
30 to 39 age	*N* = 152 *p* = 0.0001 OR = 0.590 95% CI: 0.463–0.752	*N* = 20 *p* = 0.767 OR = 0.909 95% CI: 0.574–1.438	*N* = 04 *p* = 0.832 OR = 1.287 95% CI: 0.474–3.488	*N* = 95 *p* = 0.0001 OR = 1.880 95% CI: 1.461–2.419	*N* = 00 *p* = 0.606 OR = 1.795 95% CI: 0.105–30.626	*N* = 46
40 to 49 age	*N* = 550 *p* = 0.0001 OR = 0.581 95% CI: 0.510–0.662	*N* = 70 *p* = 0.324 OR = 0.875 95% CI: 0.682–1.123	*N* = 13 *p* = 0.709 OR = 1.164 95% CI: 0.661–2.049	*N* = 341 *p* = 0.0001 OR = 1.926 95% CI: 1.680–2.208	*N* = 03 *p* = 0.099 OR = 3.646 95% CI: 1.046–12.711	*N* = 147
50 to 59 age	*N* = 1,827 *p* = 0.0001 OR = 0.768 95% CI: 0.707–0.835	*N* = 207 *p* = 0.063 OR = 0.862 95% CI: 0.739–1.004	*N* = 45 *p* = 0.034 OR = 1.455 95% CI: 1.042–2.031	*N* = 804 *p* = 0.0001 OR = 1.413 95% CI: 1.291–1.547	*N* = 04 *p* = 0.644 OR = 1.565 95% CI: 0.510–4.804	*N* = 328
60 to 69 age	*N* = 3,522 *p* = 0.0001 OR = 0.873 95% CI: 0.816–0.935	*N* = 494 *p* = 0.0001 OR = 1.258 95% CI: 1.122–1.410	*N* = 52 *p* = 0.165 OR = 0.789 95% CI: 0.575–1.084	*N* = 1,251 *p* = 0.040 OR = 1.084 95% CI: 1.004–1.170	*N* = 06 *p* = 0.854 OR = 1.254 95% CI: 0.463–3.393	*N* = 571
70 to 79 age	*N* = 3652 *p* = 0.0001 OR = 1.306 95% CI: 1.215–1.403	*N* = 429 *p* = 0.187 OR = 1.084 95% CI: 0.963–1.221	*N* = 60 *p* = 0.867 OR = 1.038 95% CI: 0.767–1.406	*N* = 913 *p* = 0.0001 OR = 0.686 95% CI: 0.632–0.745	*N* = 02 *p* = 0.199 OR = 0.329 95% CI: 0.075–1.442	*N* = 559
≥80 age	*N* = 2237 *p* = 0.0001 OR = 1.510 95% CI: 1.380–1.653	*N* = 188 *p* = 0.0002 OR = 0.735 95% CI: 0.627–0.862	*N* = 29 *p* = 0.359 OR = 0.816 95% CI: 0.549–1.211	*N* = 519 *p* = 0.0001 OR = 0.687 95% CI: 0.620–0.760	*N* = 01 *p* = 0.372 OR = 0.306 95% CI: 0.040–2.311	*N* = 266
Others*	*N* = 02	*N* = 00	*N* = 00	*N* = 01	*N* = 00	*N* = 06

**Schooling**						
	No schooling	1 to 3 years	4 to 7 years	8 to 11 years	≥12 years	others*
**Race**						
White	*N* = 937 *p* = 0.0001 OR = 0.366 95% CI: 0.332–0.403	*N* = 2545 *p* = 0.011 OR = 0.902 95% CI: 0.833–0.977	*N* = 2367 *p* = 0.001 OR = 1.144 95% CI: 1.051–1.244	*N* = 1607 *p* = 0.0001 OR = 1.470 95% CI: 1.325–1.629	*N* = 1277 *p* = 0.0001 OR = 2.6140 95% CI: 2.276–3.001	*N* =3249
Black	*N* = 257 *p* = 0.0001 OR = 1.854 95% CI: 1.597–2.152	*N* = 342 *p* = 0.129 OR = 1.112 95% CI: 0.972–1.272	*N* = 278 *p* = 0.844 OR = 0.983 95% CI: 0.852–1.133	*N* = 140 *p* = 0.001 OR = 0.731 95% CI: 0.608–0.879	*N* = 54 *p* = 0.0001 OR = 0.3735 95% CI: 0.282–0.493	*N* = 343
Yellow	*N* = 14 *p* = 0.154 OR = 0.6475 95% CI: 0.371–1.128	*N* = 33 *p* = 0.237 OR = 0.773 95% CI: 0.520–1.148	*N* = 35 *p* = 0.933 OR = 1.003 95% CI: 0.680–1.478	*N* = 21 *p* = 0.861 OR = 0.932 95% CI: 0.584–1.490	*N* = 30 *p* = 0.0002 OR = 2.1926 95% CI: 1.4549–3.304	*N* = 70
Brown	*N* = 780 *p* = 0.0001 OR = 2.458 95% CI: 2.221–2.719	*N* = 963 *p* = 0.029 OR = 1.1030 95% CI: 1.010–1.204	*N* = 733 *p* = 0.0009 OR = 0.852 95% CI: 0.775–0.935	*N* = 408 *p* = 0.0001 OR = 0.711 95% CI: 0.632–0.798	*N* = 179 *p* = 0.0001 OR = 0.3916 95% CI: 0.333–0.460	*N* = 891
Indigenous	*N* = 06 *p* = 0.0005 OR = 8,309 95% CI: 2.342–29.470	*N* = 01 *p* = 0.304 OR = 0.261 95% CI: 0.033–2.059	*N* = 03 *p* = 0.928 OR = 1.204 95% CI: 0.3111–4.658	*N* = 00 *p* = 0.320 OR = 0.237 95% CI: 0.013–4.04	*N* = 00 *p* = 0.503 OR = 0.354 95% CI: 0.020–6.05	*N* = 07
Others*	*N* = 182	*N* = 100	*N* = 85	*N* = 51	*N* = 61	*N* = 1450
**Age**						
≤19 age	*N* = 0 *p* = 0.613 OR = 0.407 95% CI: 0.022–7.241	*N* = 02 *p* = 0.826 OR = 1.220 95% CI: 0.223–6.663	*N* = 01 *p* = 0.975 OR = 0.582 95% CI: 0.068–4.989	*N* = 03 *p* = 0.091 OR = 5.161 95% CI: 1.041–25.590	*N* = 00 *p* = 0.798 OR = 0.581 95% CI: 0.032–10.327	*N* = 02
20 to 29 age	*N* = 04 *p* = 0.153 OR = 0.440 95% CI: 0.158–1.223	*N* = 13 *p* = 0.620 OR = 0.812 95% CI: 0.433–1.524	*N* = 08 *p* = 0.127 OR = 0.528 95% CI: 0.248–1.124	*N* = 16 *p* = 0.007 OR = 2.301 95% CI: 1.275–4.154	*N* = 11 *p* = 0.055 OR = 2.035 95% CI: 1.044–3.967	*N* = 23
30 to 39 age	*N* = 29 *p* = 0.288 OR = 0.793 95% CI: 0.535–1.175	*N* = 49 *p* = 0.036 OR = 0.703 95% CI: 0.510–0.968	*N* = 66 *p* = 0.172 OR = 1.237 95% CI: 0.925–1.654	*N* = 41 *p* = 0.415 OR = 1.171 95% CI: 0.831–1.648	*N* = 33 *p* = 0.166 OR = 1.326 95% CI: 0.913–1.925	*N* = 99
40 to 49 age	*N* = 94 *p* = 0.003 OR = 0.717 95% CI: 0.575–0.894	*N* = 174 *p* = 0.0001 OR = 0.690 95% CI: 0.580–0.820	*N* = 210 *p* = 0.351 OR = 1.084 95% CI: 0.921–1.276	*N* = 150 *p* = 0.019 OR = 1.250 95% CI: 1.040–1.503	*N* = 135 *p* = 0.0001 OR = 1.643 95% CI: 1.354–1.994	*N* = 361
50 to 59 age	*N* = 267 *p* = 0.0001 OR = 0.645 95% CI: 0.563–0.740	*N* = 555 *p* = 0.0001 OR = 0.729 95% CI: 0.657–0.809	*N* = 590 *p* = 0.909 OR = 0.992 95% CI: 0.896–1.099	*N* = 483 *p* = 0.0001 OR = 1.454 95% CI: 1.299–1.627	*N* = 385 *p* = 0.0001 OR = 1.663 95% CI: 1.469–1.884	*N* = 935
60 to 69 age	N = 609 p =0.015 OR = 0.881 95% CI: 0.795–0.975	N = 1,202 p = 0.978 OR = 0.998 95% CI: 0.920–1.082	N = 1,071 p = 0.571 OR = 1.025 95% CI: 0.943–1.115	N = 653 p = 0.343 OR = 0.951 95% CI: 0.861–1.052	N = 538 p = 0.001 OR = 1.196 95% CI: 1.070–1.326	N = 1,823
70 to 79 age	*N* = 683 *p* = 0.001 OR = 1.172 95% CI: 1.061–1.294	*N* = 1,227 *p* = 0.0003 OR = 1.160 95% CI: 1.070–1.258	*N* = 1,052 *p* = 0.030 OR = 1.098 95% CI: 1.009–1.194	*N* = 563 *p* = 0.0002 OR = 0.818 95% CI: 0.738–0.908	*N* = 321 *p* = 0.0001 OR = 0.592 95% CI: 0.521–0.673	*N* = 1,769
≥80 age	*N* = 489 *p* = 0.0001 OR = 1.580 95% CI: 1.411–1.768	*N* = 762 *p* = 0.0001 OR = 1.280 95% CI: 1.163–1.410	*N* = 503 *p* = 0.0001 OR = 0.797 95% CI: 0.716–0.887	*N* = 317 *p* = 0.001 OR = 0.806 95% CI: 0.709–0.916	*N* = 178 *p* = 0.0001 OR = 0.596 95% CI: 0.506–0.701	*N* = 991
Others*	*N* = 01	*N* = 00	*N* = 00	*N* = 01	*N* = 00	*N* = 07

**Status**					
	Single	Married	Widowed	Divorced	Others*
**Race**					
White	*N* = 2,165 *p* = 0.0001 OR = 0.524 95% CI: 0.486–0.565	*N* = 4,328 *p* = 0.0002 OR = 0.137 95% CI: 1.062–1.217	*N* = 4,353 *p* = 0.0001 OR = 1.365 95% CI: 1.273–1.463	*N* = 760 *p* = 0.0001 OR = 1.423 95% CI: 1.228–1.649	*N* = 376
Black	*N* = 444 *p* = 0.0001 OR = 1.779 95% CI: 1.578–2.005	*N* = 372 *p* = 0.0001 OR = 0.637 95% CI: 0.563–0.721	*N* = 477 *p* = 0.878 OR = 0.989 95% CI: 0.880–1.111	*N* = 66 *p* = 0.081 OR = 0.790 95% CI: 0.612–1.021	*N* = 55
Yellow	*N* = 45 *p* = 0.870 OR = 1.044 95% CI: 0.746–1.459	*N* = 81 *p* = 0.151 OR = 1.246 95% CI: 0.935–1.660	*N* = 62 *P* = 0.335 OR = 0.851 95% CI: 0.629–1.154	*N* = 07 *P* = 0.208 OR = 0.583 95% CI: 0.273–1.245	*N* = 08
Brown	*N* = 1,115 *p* = 0.0001 OR = 1.694 95% CI: 1.560–1.839	*N* = 1,357 *p* = 0.878 OR = 1.007 95% CI: 0.933–1.086	*N* = 1,072 *p* = 0.0001 OR = 0.684 95% CI: 0.632–0.741	*N* = 175 *p* = 0.0003 OR = 0.731 95% CI: 0.618–0.864	*N* = 235
Indigenous	*N* = 04 *p* = 0.569 OR = 1.739 95% CI: 0.523–5.780	*N* = 05 *p* = 0.935 OR = 1.250 95% CI: 0.396–3.941	*N* = 03 *p* = 0.655 OR = 0.610 95% CI: 0.165–2.255	*N* = 00 *p* = 0.792 OR = 0.629 95% CI: 0.037–10.647	*N* = 05
Others*	*N* = 356	*N* = 651	*N* = 574	*N* = 58	*N* = 290
**Age**					
≤19 years	*N* = 08 *p* = 0.0001 OR = 59.418 95% CI: 3.426–1030.4	*N* = 00 *p* = 0.074 OR = 0,101 95% CI: 0.005–1.761	*N* = 00 *p* = 0.085 OR = 0.107 95% CI: 0.006–1.868	*N* = 00 *p* = 0.484 OR = 0.962 95% CI: 0.055–16.699	*N* = 00
20 to 29 years	*N* = 40 *p* = 0.0001 OR = 5.410 95% CI: 3.297–8.874	*N* = 23 *p* = 0.858 OR = 0.923 95% CI: 0.556–1.534	*N* = 02 *p* = 0.0001 OR = 0.057 95% CI: 0.013–0.232	*N* = 01 *p* = 0.223 OR = 0.251 95% CI: 0.034–1.812	*N* = 09
30 to 39 years	*N* = 149 *p* = 0.0001 OR = 3.899 95% CI: 3.083–4.930	*N* = 109 *p* = 0.256 OR = 1.064 95% CI: 0.837–1.354	*N* = 11 *p* = 0.0001 OR = 0.112 95% CI: 0.061–0.207	*N* = 17 *p* = 0.991 OR = 1.035 95% CI: 0.631–1.697	*N* = 31
40 to 49 years	*N* = 401 *p* = 0.0001 OR = 2.355 95% CI: 2.067–2.683	*N* = 490 *p* = 0.0001 OR = 1.604 95% CI: 1.414–1.819	*N* = 63 *p* = 0.0001 OR = 0.110 95% CI: 0.085–0.143	*N* = 76 *p* = 0.025 OR = 1.328 95% CI: 1.042–1.692	*N* = 94
50 to 59 years	*N* = 898 *p* = 0.0001 OR = 1.600 95% CI: 1.467–1.746	*N* = 1,500 *p* = 0.0001 OR = 1.891 95% CI: 1.748–2.046	*N* = 392 *p* = 0.0001 OR = 0.226 95% CI: 0.202–0.252	*N* = 239 *p* = 0.0001 OR = 1.519 95% CI: 1.308–1.764	*N* = 186
60 to 69 years	*N* = 1,198 *p* = 0.030 OR = 0.918 95% CI: 0.851–0.991	*N* = 2,548 *p* = 0.0001 OR = 1.683 95% CI: 1.578–1.794	*N* = 1,484 *p* = 0.0001 OR = 0.554 95% CI: 0.517-0.594	*N* = 403 *p* = 0.0001 OR = 1.421 95% CI: 1.250–1.615	*N* = 263
70 to 79 years	*N* = 908 *p* = 0.0001 OR = 0.628 95% CI: 0.579–0.682	*N* = 1,683 *p* = 0.0001 OR = 0.719 95% CI: 0.672–0.770	*N* = 2,522 *p* = 0.0001 OR = 2.016 95% CI: 1.889–2.152	*N* = 254 *p* = 0.0002 OR = 0.755 95% CI: 0.653–0.872	*N* = 248
≥80 years	*N* = 525 *p* = 0.0001 OR = 0.667 95% CI: 0.603–0.738	*N* = 439 *p* = 0.0001 OR = 0.234 95% CI: 0.211–0.261	*N* = 2,066 *p* = 0.0001 OR = 4.859 95% CI: 4.476–5.276	*N* = 76 *p* = 0.0001 OR = 0.039 95% CI: 0.031–0.050	*N* = 134
Others*	*N* = 02	*N* = 02	*N* = 01	*N* = 00	*N* = 4
**Schooling**					
No schooling	*N* = 576 *p* = 0.0001 OR = 1.352 95% CI: 1.216–1.503	*N* = 605 *p* = 0.0001 OR = 0.672 95% CI: 0.607–0.744	*N* = 853 *p* = 0.0001 OR = 1.371 95% CI: 1.246–1.508	*N* = 61 *p* = 0.0001 OR = 0.395 95% CI: 0.303–0.515	*N* = 81
01 to 03 years	*N* = 708 *p* = 0.0001 OR = 0.679 95% CI: 0.618–0.746	*N* = 1,412 *p* = 0.997 OR = 1.001 95% CI: 0.926–1.082	*N* = 1,570 *p* = 0.0001 OR = 1.429 95% CI: 1.323–1.544	*N* = 200 *p* = 0.0002 OR = 0.729 95% CI: 0.619–0.858	*N* = 94
04 to 07 years	N = 680 p = 0.106 OR = 0.922 95% CI: 0.836–1.016	N = 1,329 p = 0.0003 OR = 1,159 95% CI: 1.070–1.256	N = 1,190 p = 0.732 OR = 1.015 95% CI: 0.935–1.102	N = 222 p = 0.839 OR = 1.020 95% CI: 0.870–1.194	N = 80
08 to 11 years	*N* = 537 *p* = 0.020 OR = 1.137 95% CI: 1.021–1.265	*N* = 824 *p* = 0.087 OR = 1.088 95% CI: 0.989–1.196	*N* = 626 *p* = 0.0001 OR = 0.731 95% CI: 0.660–0.808	*N* = 186 *p* = 0.0001 OR = 1.471 95% CI: 1.242–1.743	*N* = 54
≥12 years	*N* = 486 *p* = 0.0001 OR = 1.633 95% CI: 1.455–1.834	*N* = 597 *p* = 0.091 OR = 1.100 95% CI: 0.986–1.226	*N* = 301 *p* = 0.0001 OR = 0.414 95% CI: 0.363–0.4729	*N* = 177 *p* = 0.0001 OR = 2.097 95% CI: 1.761–2.497	*N* = 40
Others*	*N* = 1,142	*N* = 2,027	*N* = 2,001	*N* = 220	*N* = 01

* Category not defined.
